# Development of the maize 5.5K loci panel for genomic prediction through genotyping by target sequencing

**DOI:** 10.3389/fpls.2022.972791

**Published:** 2022-11-11

**Authors:** Juan Ma, Yanyong Cao, Yanzhao Wang, Yong Ding

**Affiliations:** Institute of Cereal Crops, Henan Academy of Agricultural Sciences, Zhengzhou, China

**Keywords:** maize, genotyping by target sequencing, genomic prediction, hybrid prediction, general combining ability

## Abstract

Genotyping platforms are important for genetic research and molecular breeding. In this study, a low-density genotyping platform containing 5.5K SNP markers was successfully developed in maize using genotyping by target sequencing (GBTS) technology with capture-in-solution. Two maize populations (Pop1 and Pop2) were used to validate the GBTS panel for genetic and molecular breeding studies. Pop1 comprised 942 hybrids derived from 250 inbred lines and four testers, and Pop2 contained 540 hybrids which were generated from 123 new-developed inbred lines and eight testers. The genetic analyses showed that the average polymorphic information content and genetic diversity values ranged from 0.27 to 0.38 in both populations using all filtered genotyping data. The mean missing rate was 1.23% across populations. The Structure and UPGMA tree analyses revealed similar genetic divergences (76-89%) in both populations. Genomic prediction analyses showed that the prediction accuracy of reproducing kernel Hilbert space (RKHS) was slightly lower than that of genomic best linear unbiased prediction (GBLUP) and three Bayesian methods for general combining ability of grain yield per plant and three yield-related traits in both populations, whereas RKHS with additive effects showed superior advantages over the other four methods in Pop1. In Pop1, the GBLUP and three Bayesian methods with additive-dominance model improved the prediction accuracies by 4.89-134.52% for the four traits in comparison to the additive model. In Pop2, the inclusion of dominance did not improve the accuracy in most cases. In general, low accuracies (0.33-0.43) were achieved for general combing ability of the four traits in Pop1, whereas moderate-to-high accuracies (0.52-0.65) were observed in Pop2. For hybrid performance prediction, the accuracies were moderate to high (0.51-0.75) for the four traits in both populations using the additive-dominance model. This study suggests a reliable genotyping platform that can be implemented in genomic selection-assisted breeding to accelerate maize new cultivar development and improvement.

## Introduction

Genotyping platforms are prerequisite for genomic research, genetic analysis, and marker-assisted breeding in animals and plants. Compared with other marker types, single nucleotide polymorphisms (SNPs), as the most extensive and stable genomic variations of multiple species, are ideal markers for genotyping because of their advantages in ultra-high-throughput detection and easy integration of genotypic data (Zhang et al., 2020a).

Array-based and sequencing-based technologies (next-generation sequencing) are the major genotyping platforms which are available for the screening of SNP markers. In the former technology, the fixed nature of SNPs on an array is helpful for cross-project comparisons because the same markers are used (Rasheed et al., 2017). However, when new SNPs are required, the array-based genotyping platform can be expensive because the array must be redesigned (Rasheed et al., 2017). Sequencing-based technologies contain three strategies to obtain SNP markers. Whole genome resequencing, identifying all sequence variability, is still high-cost for genotyping large populations with the aim to perform genetic and breeding studies. Reduced-representation genome sequencing (restriction-site associated DNA and genotyping-by-sequencing), a partial or selective sequencing, is simple, quick, and low-cost (Davey et al., 2011; Andrews et al., 2016). The two strategies may not allow comparisons across projects because different sequencing technologies and analysis pipelines affect the selection of SNPs detected (Torkamaneh et al., 2016; Burridge et al., 2018).

Genotyping by target sequencing (GBTS), a newly developed sequencing-based genotyping platform, involves the capture of target genomic loci by probes (Guo et al., 2021). GBTS integrates the advantages of array-based and partial sequencing, and possesses the characteristics of customized flexibility, high throughout, and low cost (Guo et al., 2019). The technology also allows cross-project comparisons due to the target genomic loci. GBTS mainly contains multiplex PCR-based (GenoPlexs) (Zhang et al., 2020a) and probe-in-solution-based target sequencing (GenoBaits) (Guo et al., 2019). Recently, Guo et al. (2021) improved the latter system and developed a multiple SNP (mSNP) approach where mSNPs can be captured from a single amplicon. GBTS has been successfully utilized for genotyping, genetic diversity analysis, quantitative trait locus mapping, genome-wide association study, and traditional marker-assisted selection in wheat (Burridge et al., 2018), maize (Guo et al., 2019; Guo et al., 2021), pepper (Du et al., 2019), cucumber (Zhang et al., 2020a), faba bean (Wang et al., 2021), and broccoli (Shen et al., 2021). However, the application of GBTS in genomic prediction for parent and hybrid performance was rarely reported.

Hybrid breeding plays a great role in improving maize and many other crops. It mainly involves the development of inbred lines with high general combining ability (GCA) and specific combining ability (SCA) and the identification of hybrids with high yield potentials (Zhang et al., 2022). The estimation of GCA and SCA needs to conduct multi-environment trials using specific mating designs, such as the diallel cross and North Carolina II design. Therefore, the process of hybrid breeding not only requires a vast of field resources to evaluate the performances of all possible combinations among many inbred lines, but laborious work for the identification of hybrid performance. In fact, only a small proportion of crosses can be tested in the field and abundant crosses with potentials may not have the chance to be evaluated.

Genomic selection, first proposed by Meuwissen et al. (2001), aims to estimate breeding values of untested populations only having genotyping data and select inbred lines or hybrids with high yield potentials based on the information of training population which is genotyped and phenotyped. The application of GS in hybrid breeding projects can help predict the performance of untested crosses and conduct selections with the aid of genotyping platforms according to the genotypic and phenotypic information of tested populations, which can accelerate the breeding process of developing high GCA parental lines and high-yielding hybrids. The genomic prediction for combining ability and hybrid performance has been reported in maize (de Oliveira et al., 2020; Zhang et al., 2022), rice (Cui et al., 2020), wheat (Zhao et al., 2015), sorghum (Ishimori et al., 2020), and canola (Knoch et al., 2021) using genotypic data derived from the array-based and partial sequencing-based genotyping platforms. However, theses genotyping platforms are still high-cost for GS-assisted breeding programs although moderate-to-high prediction accuracies were revealed in those studies, which may guarantee a reliable prediction for the performance of unevaluated lines.

Although several SNP genotyping platforms were developed through GBTS in maize, no GBTS system was evaluated in genomic selection for GCA and hybrid performance. In addition, the current GBTS platform still needed to be specifically customized and optimized according to different applications in genetic and molecular breeding. In the present study, we designed a low-density GBTS panel from diverse resources and evaluated its applications in genotyping, population structure classification, and genomic prediction for GCA and hybrid performance.

## Materials and methods

### Design of the maize GBTS-based 5.5K loci panel

To build a reliable and genome-wide genotyping array, we selected 5,521 target SNPs from diverse resources (**Table 1**). In a previous study (Wang et al., 2022), four inbred lines Zheng58, Chang7-2, Zheng588, and ZhengH71 were sequenced through the whole genome resequencing technology (Dataset I), and the four inbred lines and their F_1_ hybrids (Zhengdan958, Zhengdan1002, Zheng588/Chang7-2, and Zheng58/ZhengH71) were sequenced through RNA-seq technology at seven seed developmental stages (Dataset II). A total of 1,973 heterosis-related SNPs were selected from the above data using two following criteria: (1) SNPs were retained when the corresponding genes were significantly differentially expressed between F_1_ hybrids and one of their corresponding parents in at least one F_1_ hybrid and one developmental stage using edgeR (log_2_|fold change| > 1, FDR< 0.05), and these differentially expressed genes were significantly correlated with the mid-parent heterosis of hundred-kernel weight (HKW) and fresh HKW in at least one developmental stage using weighted gene co-expression network analysis (**Supplementary Figure 1**), and (2) SNPs showed allele-specific expression in at least one F_1_ hybrid were selected according to the method of a previous study (Shao et al., 2019). Allele-specific expression, the imbalance between expression levels of two parental alleles in a hybrid, has been considered as a mechanism of heterosis (Shao et al., 2019). We found 653 SNPs were related with mid-parent heterosis, 1,772 SNPs were considered allele-specific expression, and 452 SNPs were passed the both selected criteria.

**Table 1:** The number of target SNPs selected from different resources.

Category^#^	Number
Heterosis-related SNPs (Dataset I and II)	1,973
Published references	487
113 known genes (Dataset II)	184
Dataset I	898
Dataset II	776
Dataset III	836
maize GBTS-based 48K loci panel	367

^#^ Dataset I: the whole genome resequencing data of four inbred lines Zheng58, Chang7-2, Zheng588, and ZhengH71; Dataset II: the RNA-seq data of Zheng58, Chang7-2, Zheng588, and ZhengH71 and their F_1_ hybrids (Zhengdan958, Zhengdan1002, Zheng588/Chang7-2, and Zheng58/ZhengH71) at seven seed developmental stages; Dataset III: the RNA-seq data of Qi319, Ye478, B104, AJ525, A350, A314, and LH209 in terms of multiple tissues containing roots, leaves, seeds, and young ears.

**Figure 1:**
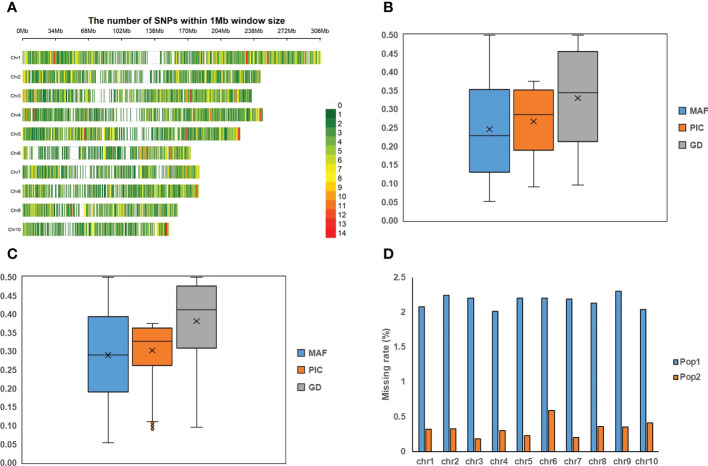
The information of 5.5K loci panel and the genotyping profiles in two populations. **(A)** The distributions of 5.5K target markers on ten chromosomes. **(B, C)** represent the distributions of minor allele frequency (MAF), polymorphic information content (PIC), and gene diversity (GD) in Pop1 and Pop2, respectively. **(D)** The missing rate on ten chromosomes in both populations.

A great number of genes have been reported to regulate maize development and related agronomic traits. To cover these functional genetic loci, we included 184 synonymous SNPs or SNPs located at exonic or UTR regions from 113 known genes based on the Dataset II (**Table 1**, **Supplementary Table 1**). In addition, 487 SNPs were selected from important loci associated with grain yield and yield-related traits from published references (Yang et al., 2014; Liu et al., 2017a; Zhang et al., 2017; Pang et al., 2019; Liu et al., 2020; Ma et al., 2021; Ma and Cao, 2021). To make markers as evenly distribute across the genome as possible, 898 SNPs were selected from Dataset I, 776 SNPs were selected from Dataset II, 836 SNPs were selected from the RNA-seq data of Qi319, Ye478, B104, AJ525, A350, A314, and LH209 in terms of multiple tissues containing roots, leaves, seeds, and young ears (Dataset III), and 367 SNPs were selected from maize GBTS-based 48K loci panel (China Golden Marker, Beijing) after the quality control of GC content (40-60%) and the filtration of multi-copy SNPs. The distribution of 5.5K target markers on ten chromosomes was demonstrated in **Figure 1A**.

### Plant materials, field trials, and evaluation of agronomic traits

To verify the effectiveness of the low-density GBTS genotyping platform, two populations (Pop1 and Pop2) were used in the present study. Pop1, a genetic population, contained 254 inbred lines from China (150) and USA (104), of which 250 inbred lines and four testers (Zheng58, Chang7-2, PH6WC, and PH4CV) produced 942 F_1_ hybrids using North Carolina II mating design. Pop2, a breeding population, consisted of 123 new-developed inbred lines and eight testers (Chang7-2, PH4CV, Nongxi531, M119, M189, 20H1419, L119A, and S110T), which generated 540 F_1_ hybrids. Two hybrid populations were evaluated in field experiments at Xinxiang and Zhoukou, Henan, but Pop1 and Pop2 were grown in 2020 and 2021, respectively. Entries were evaluated in one-row plot using randomized complete block design with two replicates. The plot size was 4 m and 3.3 m in length in Xinxiang and Zhoukou, respectively, all with 0.60 m between rows and 0.22 m between plants. Traits determined were grain yield per plant (GYP), ear weight (EW), HKW, and kernel number per row (KNR) in both populations. The analysis of variance was calculated following a linear mixed model.

*y*=*μ*+*E*+*R*+*GCA*_*L*_+*GCA*_*T*_+*SCA*+*GCA*_*L*_×*E*+*GCA*_*T*_×*E*+*SCA*×*E*+*ϵ* , where *y* indicates the phenotypic value of hybrids, *μ* denotes the overall mean, *E* represents environment effect, *R* represents replicates. *GCA*_*L*_ and *GCA*_*T*_ are effects of inbred lines and testers, respectively; *SCA* is the effect of the combinations of inbred lines and testers; *GCA*_*L*_×*E* , *GCA*_*T*_×*E* , and *SCA*×*E* indicate *GCA*_*L*_ , *GCA*_*T*_ , and *SCA* interaction effects with environment, respectively. The variance components and GCA effects were calculated using R package lme4. The heritability of GCA effects was calculated using the following formula which was modified from Liu et al. (2021).

$H_{GCA}^{2}=\frac{\sigma_{GCA_{L}}^{2}+\sigma_{GCA_{T}}^{2}}{\sigma_{GCA_{L}}^{2}+\sigma_{GCA_{T}}^{2}+\frac{\sigma_{SCA}^{2}}{t}+\frac{\sigma_{GCA_{L\times E}}^{2}}{e}+\frac{\sigma_{GCA_{T\times E}}^{2}}{e}+\frac{\sigma_{SCA\times E}^{2}}{te}+\frac{\sigma_{\epsilon }^{2}}{ter}}$ , where $\sigma_{GCA_{L}}^{2},\sigma_{GCA_{T}}^{2},\sigma_{SCA}^{2},\sigma_{GCA_{L\times E}}^{2},\sigma_{GCA_{T\times E}}^{2},\sigma_{SCA\times E}^{2}$ , and $\sigma_{\epsilon }^{2}$ represent the GCA variance of inbred lines, the GCA variance of testers, the variance of SCA, the interaction variance between the GCA of inbred lines and environment, the interaction variance between the GCA of testers and environment, the interaction variance between SCA and environment, and residual variance, respectively, and *t* , *r* , and *e* are the numbers of tester lines, replicates, and environments, respectively. Heritability at per mean level and best linear unbiased estimate (BLUE) values of hybrid traits were calculated using QTL IciMapping v4.2 software (Meng et al., 2015).

### Genotyping and analyses based on the maize 5.5K loci panel

All parental lines of the two populations were used for genotyping. CTAB method was adopted to extract genomic DNA from fresh leaves. The length of each probe for the 5.5K loci panel was 100 bp to cover the SNP regions, which can capture approximately 250-400 bp sequence. The major processes of GBTS based on liquid-phase probe hybridization were as follows according to Wang et al. (2021): (1) Genomic DNA was fragmented and added a sequencing adapter, (2) The biotin-labelled RNA probe was combined with the DNA fragments that had already been attached to the adapter sequence, (3) Streptavidin-coated magnetic beads were combined with the double stranded complex of biotin-labelled RNA probe and DNA (probe excess), (4) Washing to obtain the DNA of the target region to remove nonspecific hybridization and improve the capture efficiency, and (5) The eluted DNA products were amplified by PCR and sequenced using Illumina NovaSeq 6000 platform (China Golden Marker, Beijing). BWA software was used to align the filtered reads to B73 RefGen v4 (http://www.gramene.org/). GATK v4.1.2.0 (McKenna et al., 2010) was used to detect variants. Vcftools and PLINK software were used to filter minDP< 11, minGQ< 20, minor allele frequency (MAF) ≤ 0.05, missing rate > 10%, and heterozygous rate > 1%. The polymorphic information content (PIC) was calculated according to the following equation that was proposed by Botstein et al. (1980).

$PIC=1-(\sum_{i=1}^{n}P_{i}^{2})-\sum_{i=1}^{n-1}\sum_{j=i+1}^{n}2P_{i}^{2}P_{j}^{2}$ , where *P*_*i*_ and *P*_*j*_ are the population frequencies of the *i*th and the *j*th allele. Gene diversity (GD) was estimated as:


$$GD=1-(\sum_{i=1}^{n}P_{i}^{2})$$

### Population structure analysis

All filtered genotyping data were used for population structure analysis. Population structure was inferred using the Bayesian Markov Chain Monte Carlo (MCMC) program in Structure v2.3.4 (Pritchard et al., 2000). The number of subgroups (*K*) was set from 1 to 8 in Pop1, whereas that was 1 to 10 in Pop2.The length of burnin period and the number of MCMC replicates after burnin were 5,000 and 50,000, respectively. The Structure output was visualized by Structure Harvester (Earl and vonHoldt, 2012), and delta *K* was used to determine the optimal number of subgroups. The FullSearch algorithm in CLUMPP v1.1.2 (Jakobsson and Rosenberg, 2007) was used to estimate cluster membership coeficient matrices from the optimal subgroup. To verify the optimal number of clusters, unweighted pair-group method with arithmetic means (UPGMA) tree was performed using the software TASSEL v5.2.60 (Bradbury et al., 2007). The circular tree was demonstrated using R package ggtree.

### Genomic prediction for general combining ability and hybrid performance

Five models including Bayes A, Bayes C, Bayesian least absolute shrinkage and selection operator (Bayesian LASSO), genomic best linear unbiased prediction (GBLUP), and reproducing kernel Hilbert space (RKHS) were adopted for genomic prediction using all filtered genotyping data. For RKHS, three kernels were used and their bandwidth parameter *h* was set at 0.1, 0.5, and 2.5. For the GCA prediction, the genotypes were coded by -1 for one homozygote, 0 for the heterozygote, and 1 for the other homozygote. Randomized imputation was used for missing markers, according to the known genotype frequency (Ma and Cao, 2021). The above five GS methods were used to perform hybrid phenotypic prediction using additive (A) and additive plus dominance (AD) model. In the A model, the homozygous genotypes were coded as -1 and 1, and the heterozygous genotypes were coded as 0. For the mating type A1A1 × A1A2 and A2A2 × A1A2, these hybrids were coded as -0.5 and 0.5, respectively. When the mating type was A1A2 × A1A2, their hybrids were coded as 0. For the dominance model, the homozygous genotypes were coded as 0, and the heterozygous genotypes were coded as 1. For the mating type A1A1 × A1A2 and A2A2 × A1A2, the hybrids were all coded as 0.5.

All GS models and prediction strategies were performed using the R package, BGLR (Pérez and de los Campos, 2014). For all models, the number of Gibbs iterations was 12,000, and the burn-in was 3,000. A 10-fold cross-validation scheme was used and repeated 100 times for all prediction methods and models. In the 10-fold cross validation, 90% inbred lines or hybrids were selected as the training set to predict the remaining 10% inbred lines or hybrids as the testing set. The average correlation coefficient between genomic estimated breeding values and phenotypic values in the testing set was used to estimate the accuracies of different GS models.

### Genomic prediction across projects

The fixed nature of the GBTS technology allowed the comparisons between different projects. The genotyping of the two populations was conducted in two batches, therefore we found common SNPs between the filtered genotyping data of both populations. Based on these common SNPs, the prediction accuracy of GCA and hybrid performance was calculated using the RKHS method. As all filtered genotyping data, the same cross-validation and parameters were used.

### Genomic prediction for potentially functional markers

Among the 5,521 target SNPs, 2,644 SNPs were identified from the weighted gene co-expression network analysis, allele-specific expression analysis, known genes, and published references (**Table 1**), which were defined as potentially functional markers. These functional SNPs existed in the filtered genotyping data were used as marker subset to predict GCA and hybrid performance using the RKHS method. To validate the performance of these markers, the same number of other target SNPs was also used to conduct the genomic prediction.

## Results

### The performance of hybrids and general combining ability

Hybrid phenotypes and parental GCA were analyzed in this study. Descriptive statistics were shown in **Supplementary Figure 2**. Genetic correlations showed GYP and EW showed positive and high correlations in both hybrid populations, with *r* value ranging from 0.98 to 0.99 (**Supplementary Figure 3**). HKW and KNR had low or no correlations, but they were positively and significantly correlated with GYP and EW, with *r* value ranging from 0.36 to 0.64 in both hybrid populations. As the hybrid trait *per se*, high correlations (*r* = 0.98) were also observed between the GCA effects of GYP and EW in the two populations. For GCA effects of other traits, similar correlation values were found as those of the hybrid traits.

Analysis of variance showed that significant GCA, SCA, GCA-by-environment interaction, and SCA-by-environment interaction variances were revealed for all traits except for one source in KNR of Pop1 (**Supplementary Table 2**). In Pop1, the variances of SCA were much higher than those of GCA for GYP and EW. The contrast trend was observed in Pop2. The heritabilities of traits in hybrids ranged from 0.55 for KNR in Pop2 to 0.71 for HKW in Pop1. In Pop1, the heritabilities of GCA effects for GYP and EW were low (*H* ^2^ = 0.16-0.17), whereas those were high (*H* ^2^ = 0.82-0.84) in Pop2. For other traits, the heritabilities of GCA effects ranged from 0.50 to 0.84. Big variations observed in the heritabilities of GCA for GYP and EW across populations could be attributed to genetic backgrounds, environmental effects, or the interactions between them.

### Profiles of genotyping using the maize GBTS-based 5.5K loci panel

Based on the 5,521 target SNPs using the GBTS technology with capture-in-solution, 75,876 and 33,971 raw SNPs were detected in Pop1 and Pop2, respectively. After filtering, 20,210 and 11,734 high-quality SNPs were generated for Pop1 and Pop2, respectively, which were used for the further genetic analyses. The number of SNPs per chromosome ranged from 1,383 (chromosome 10) to 3,446 (chromosome 1) in Pop1, whereas that ranged from 376 (chromosome 6) to 2,391 (chromosome 1) in Pop2 (**Supplementary Figure 4A**). The mean MAF after filtering across all SNPs was 0.25 and 0.29 in Pop1 and Pop2, respectively (**Figures 1B, C**). The mean missing rate after filtering across all SNPs was 2.16% and 0.30% in Pop1 and Pop2, respectively (**Figure 1D**). The average PIC and GD values were 0.27 and 0.33 in Pop1, respectively, whereas those of Pop2 were 0.30 and 0.38, respectively. Among these filtered markers, 4,865 and 2,237 target SNPs were found in Pop1 and Pop2, respectively (**Supplementary Figure 5A**). The mean MAF, PIC, and GD values of target SNPs were slightly higher than those of all filtered markers in both populations (**Figures 1B, C**, **Supplementary Figures 5B, C**). The mean missing rate of the target SNPs was 0.12-0.87%, which was much lower than that of the full set of filtered markers (**Figure 1D** and **Supplementary Figure 5D**). These results highlighted the high quality of those target SNPs.

### Genetic structures of two maize populations

**Figure 2:**
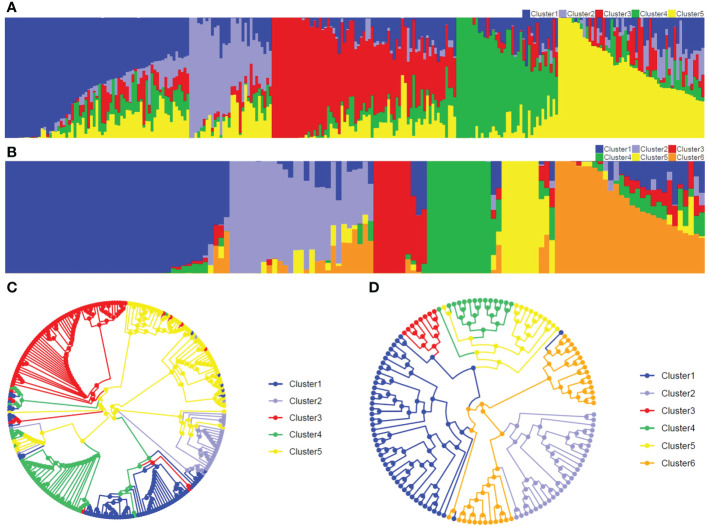
Population structure and UPGMA tree in two populations. **(A, B)** represent population structure in Pop1 (*K*=5) and Pop2 (*K*=6), respectively. Abscissa and ordinate represent the inbred lines and the membership percentage of inbred line, respectively. **(C, D)** represent circular UPGMA tree in Pop1 and Pop2, respectively.

The Structure and CLUMMP analyses revealed that Pop1 and Pop2 were divided into five and six sub-populations, respectively, based on the optimal number of *K* (**Supplementary Figure 6**). In Pop1, Cluster 1 and Cluster 2 mainly belonged to non-Stiff Stalk, Cluster 3 mainly represented Stiff Stalk, Cluster 4 indicated Tang Si Ping Tou, and Cluster 5 inferred as modified Reid group (**Figure 2A**). For the breeding population Pop2, the number of inbred lines within sub-populations ranged from 10 (Cluster 3) to 42 (Cluster 1) (**Figure 2B**). Similar genetic divergences (76-89%) were also observed using the circular UPGMA tree in both populations (**Figure 2**), which indicated that the GBTS-based 5.5K loci panel can be used for genetic analyses and assisted for the inference of germplasm origins.

### Prediction accuracies for general combining ability and hybrid performance

**Figure 3:**
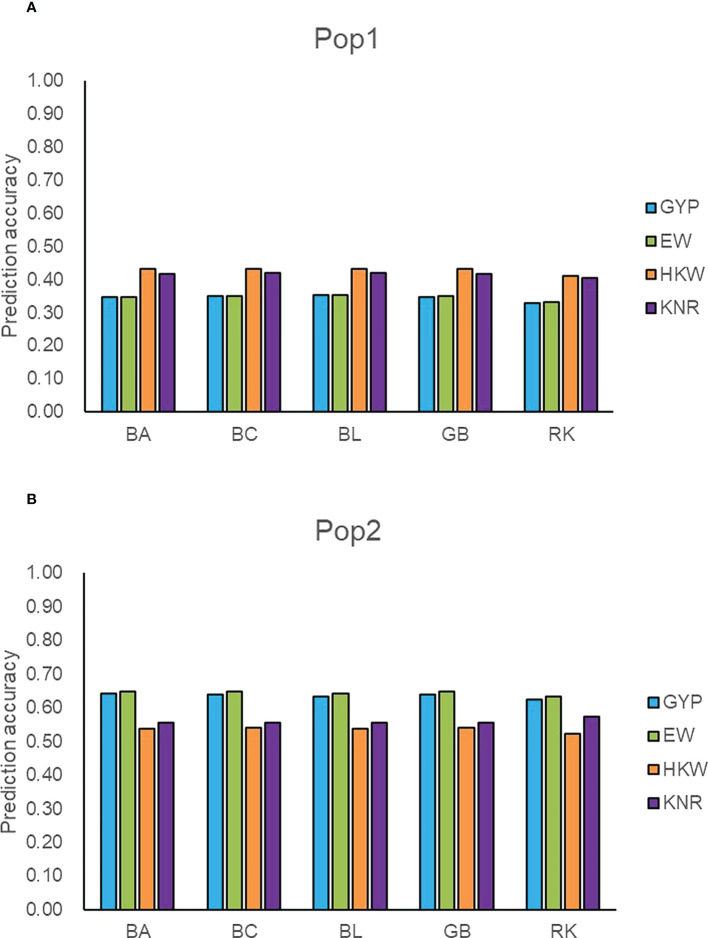
Accuracy of five models predicting general combining ability for four traits in two populations. **(A)** The prediction accuracy of GYP, EW, HKW, and KNR in Pop1. **(B)** The prediction accuracy of GYP, EW, HKW, and KNR in Pop2. GYP, EW, HKW, and KNR are abbreviations of grain yield per plant, ear weight, thousand-kernel weight, and kernel row number, respectively. BA, BC, BL, GB, and RK denote Bayes A, Bayes C, Bayesian LASSO, GBLUP and RKHS, respectively.

To detect the prediction power of the GBTS panel, we used the classical parameter model GBLUP, three Bayesian models, and a semi-parameter model RKHS to predict GCA effects of the observed traits within populations. The prediction accuracies ranged from 0.33 (GYP) to 0.43 (HKW) in Pop1, whereas those varied from 0.52 (HKW) to 0.65 (EW) in Pop2 (**Figure 3**). Regardless of populations and traits, GBLUP and the three Bayesian methods resulted in similar predictive performance for GCA effects, with the difference values ranging from 0 to 0.01. In general, the prediction accuracy of RKHS was slightly lower than that of the other four methods, with the percentage decrease ranging from 1.18 to 6.96%. Moderate-to-high accuracies were obtained for GCA effects in the breeding population (Pop2), which suggested that the maize GBTS-based 5.5K loci panel can be used for GS-assisted selection for high GCA lines.

**Figure 4:**
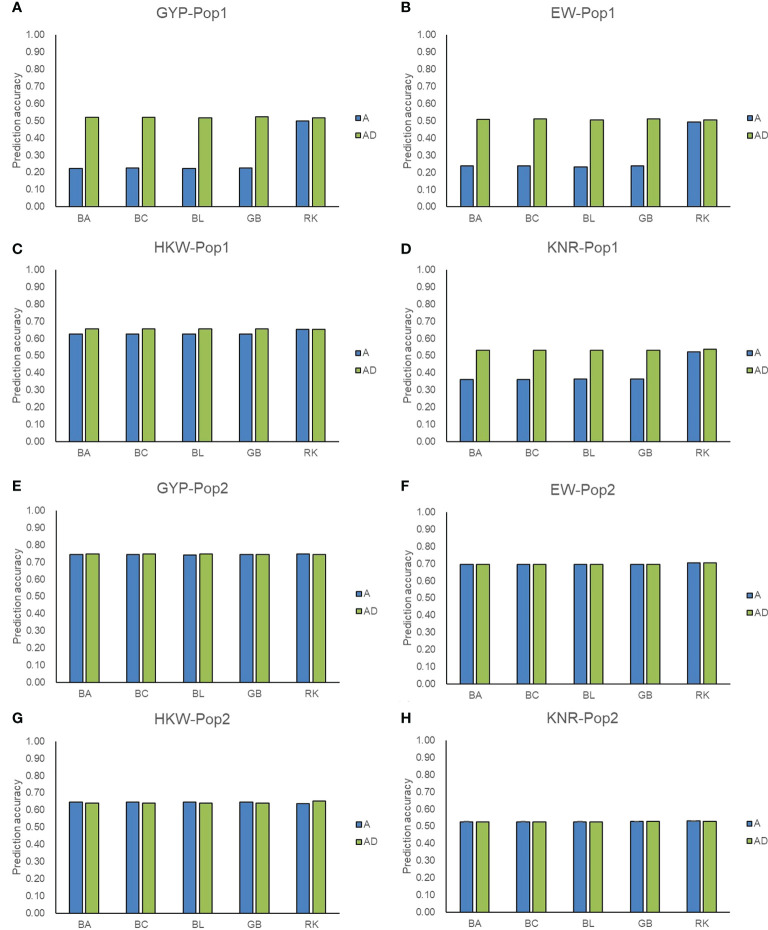
Accuracy of additive and additive-dominance model predicting hybrid performance. **(A–D)** represent the prediction accuracy for GYP, EW, HKW, and KNR in Pop1, respectively. **(E–H)** represent the prediction accuracy for GYP, EW, HKW, and KNR in Pop2, respectively. A and AD represent additive and additive-dominance model, respectively.

Except for GCA effects, the predict power of hybrid phenotypes was also evaluated for the genotyping panel. The above five GS methods incorporating additive effect only and additive plus dominance effect were adopted. In Pop1, the prediction accuracies for the performance of hybrids ranged from 0.22 (GYP) to 0.65 (HKW) in the five methods including additive effects, whereas those were improved and ranged from 0.51 (EW) to 0.66 (HKW) in the AD model (**Figure 4**). Compared with the A model, the AD model of GBLUP and the three Bayesian methods improved the accuracy by 114.35-134.52% for GYP and EW in Pop1. Compared with the A model, the percentage increase ranged from 45.66 to 46.82% for KNR when the dominance was incorporated into GBLUP and the three Bayesian methods, whereas a small percentage increase (4.89-5.15%) was observed for HKW. For RKHS, the addition of dominance slightly improved the accuracies of hybrid performance for GYP, EW, and KNR in Pop1. Due to high accuracies (0.53-0.75) achieved in the A model for all four traits in Pop2, the incorporation of dominance did not improve the accuracies of hybrid performance in most scenarios. For the A model only, RKHS showed the superior performance over the other four methods, improving the accuracy by 42.68-126.44% for GYP, EW, and KNR in Pop1. Regardless of GS methods and models, moderate-to-high accuracy values were achieved for the hybrid prediction in both populations, which supported the reliability of GS-assisted selection of excellent hybrids using the maize 5.5K loci genotyping panel.

### Cross-project comparisons and prediction accuracy for potentially functional markers

Based on all filtered SNPs, the RKHS method showed superior performance over the other four methods particularly when the additive effect was considered in Pop1 and showed similar or slightly lower advantages in other circumstances, therefore the method was used for the cross-project comparisons and the prediction for potentially functional markers. Due to the fixed nature of target genomic loci, 7,743 SNPs were simultaneously detected between 20,210 and 11,734 SNPs, accounting for 38-66% of those filtered genotyping data (**Supplementary Figure 4B**). The prediction accuracies of 7,743 SNPs ranged from 0.35 to 0.64 for GCA effects of GYP and EW in the two populations, which was slightly higher than those of all filtered markers (**Figure 3** and **Supplementary Figure 7A**). Compared with all filtered SNPs, slightly lower accuracy was found in GCA effects of HKW and KNR in Pop1. The 7,743 SNPs enabled high accuracy (0.71) for GYP with the A and AD model in Pop2, although the value was smaller than that of all markers (**Figure 4E** and **Supplementary Figure 7C**). For other circumstances, similar prediction abilities were observed between common SNPs and the full set of markers. These findings agreed with Spindel et al. (2015) who pointed out that ~ 7,000 (approximately 1 SNP for every 0.2 cM) SNPs were sufficient for GS. These suggested that the GBTS-based 5.5K loci genotyping platform can be used for cross-project comparisons in terms of genomic prediction.

**Figure 5:**
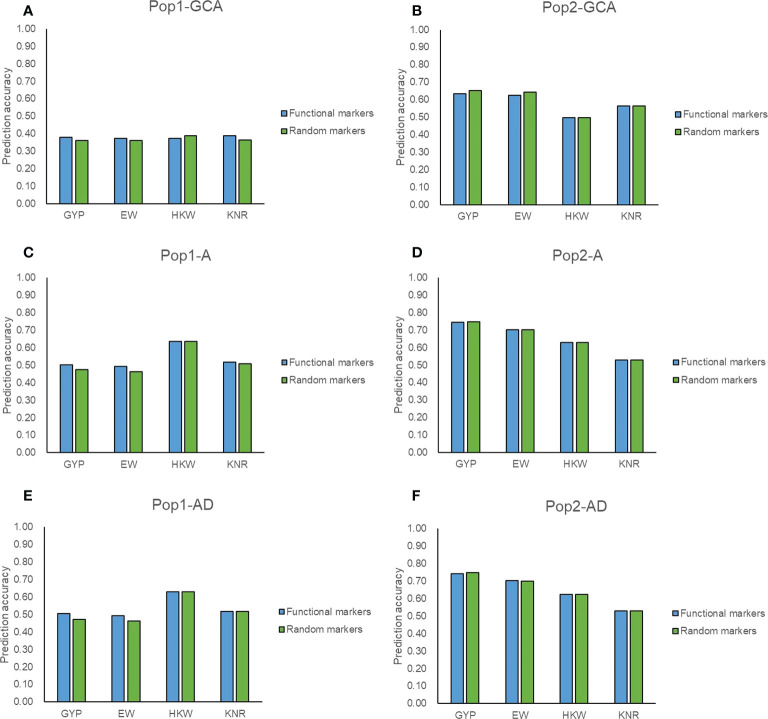
Accuracy of target SNPs in two populations. **(A, B)** represent the general combining ability prediction using RKHS method in Pop1 and Pop2, respectively. **(C, D)** represent hybrid performance prediction using RKHS with additive model (A) in Pop1 and Pop2, respectively. **(E, F)** represent hybrid performance prediction using RKHS with additive-dominance model (AD) in Pop1 and Pop2, respectively. Functional markers represent 906 target SNPs associated with mid-parent heterosis, allele-specific expression, known genes, and yield-related traits. Random markers represent 906 target SNPs which are randomly selected from 1,115 target SNPs.

Among the 5,521 target SNPs, 2,644 SNPs were associated with mid-parent heterosis, allele-specific expression, known genes, and yield-related traits and were considered as potentially functional markers. A total of 2,021 target SNPs were overlapped between the two populations among the filtered genotyping data (**Supplementary Figure 5A**), of which 906 target SNPs were potentially functional markers. The prediction ability was compared when these potentially functional markers and the same number of other target SNPs were used for genomic prediction. For GYP and EW, the prediction accuracy of potentially functional markers was consistently higher that of randomly selected markers in terms of GCA and hybrid performance in Pop1 (**Figures 5A, C, E**). The good performance was also observed for GCA prediction in both populations and for hybrid prediction with the A model in Pop1 for KNR (**Figures 5A–C**). These might prove that the selection strategies for functional SNPs were valid. Zhang et al. (2020b) found that the employment of functional genes information such as the number of favorable alleles and genotypes enabled accurately predicting maize yield. However, these potentially functional markers did not show any advantage in the remaining scenarios (**Figure 5**). Most of these target SNPs achieved similar accuracies as the full set of filtered SNPs (**Figures 3**–**5**), which again highlighted the reliability of target SNPs.

## Discussion

### Evaluation of genotyping, genetic diversity, and population structure

High-throughput genotyping technology is very important for effective crop breeding programs. GBTS technology integrates the advantages of array-based and partial sequencing, showing advantages in customized flexibility, high throughput, and low cost. In maize, a series of high-quality GBTS panels, including 1-20K SNP (GenoBaits) and 1-40K mSNP, were developed, which made the technology an effective and efficient tool for genotyping and population structure classification (Guo et al., 2019; Guo et al., 2021). In the present study, the GBTS-based 5.5K loci panel was developed mainly from the whole genome resequencing and transcriptome sequencing of Huanghuaihai maize germplasms. Two populations containing 383 accessions were genotyped using the platform. Due to the target region sequencing, the number of detected raw SNPs was approximately 14-fold and six-fold as that of the target SNPs for Pop1 and Pop2, respectively. Wang et al. (2021) found that 1,579,411 SNPs were identified and further filtered according to the Faba_bean_130K targeted next-generation sequencing genotyping platform. These all showed that the GBTS technology can detect a large number of SNPs in comparison to the capacity of target SNPs. Like the genotyping-by-sequencing or restriction-site associated DNA, the GBTS was based on next-generation sequencing, therefore the genotyping results were affected by the size of restriction fragment length, population background, and population size when samples were genotyped in different batches. Based on the filtered genotyping data, the mean missing rate across populations was 1.23%, which was lower than that of populations genotyped using GBTS-based 1-20K panels (Guo et al., 2019). The average PIC and GD values ranged from 0.27 to 0.38, which could be considered high. The biallelic nature of the SNP markers limited the range of PIC and GD values from 0 to 0.5 (Eltaher et al., 2018; Shen et al., 2021). All these proved that the GBTS-based 5.5K loci panel is available for identifying the genetic diversity of maize germplasms.

For Pop1, 254 maize accessions were divided into five subpopulations by the Structure analysis, 76% of which was in agreement with the phylogenetic tree (**Figures 2A, C**). For Pop2, Structure and phylogenetic tree results agreed with each other with 11% exceptions (**Figures 2B, D**). For Structure analysis, a sub-population membership percentage was produced and the highest percentage was used to assign one individual to one group, whereas a fixed branch position was assigned to each accession for UPGMA analysis (Wang et al., 2009; Shen et al., 2021). This discrepancy between the two methods of grouping might result in some biases. The similar results showed that the GBTS-based 5.5K loci panel can be assisted for the population structure classification.

### Potential applications in genomic selection

GS has obvious advantages for improving genetic gains in animal and plant breeding, but the price of genotyping can be prohibitive for many species (Kriaridou et al., 2020). Therefore, the development of cost-effective and user-friendly genotyping platform that is suitable for genomic selection is valuable for breeding programs with limited funds and resources. We demonstrated the potential of the GBTS-based 5.5K loci panel in genomic prediction in terms of GCA and hybrid performance using one genetic population and one breeding population. Regardless of GCA or hybrid performance prediction, higher accuracies were observed in Pop2 than in Pop1, especially for GYP and EW. The phenomena also occurred even if only common SNPs, such as 7,743 overlapped SNPs, 906 functional target SNPs, and randomly selected target SNPs, were used for prediction. Obvious variations in prediction accuracies for hybrid performance were also revealed in different maize breeding populations (Windhausen et al., 2012; Li et al., 2021). The variances of GCA and SCA varied across populations indicated that the genetic structure of GYP and EW was very different in two populations. Therefore, the differences in genetic basis of traits among different populations might be an important factor influencing the prediction accuracies (Li et al., 2021). The significant environment effect and genotype-by-environment effect were revealed across traits and populations through analysis of variance, indicating that the environment factor might affect the prediction results because the two populations were grown in different years.

Decomposing of variances of hybrid performance into GCA and SCA variances could reflect the role of additive and non-additive effects. In Pop1, the inclusion of the dominance effects could effectively improve the prediction accuracy of hybrid phenotypes in GBLUP and the three Bayesian methods. In particular, the AD model boosted the prediction accuracy for GYP and EW by more than two-fold compared with the A model, which agreed with a previous study where GBLUP with AD effects doubled the predictive capacity for maize grain yield under water-stressed trial in comparison to the A model (Dias et al., 2018). In general, these results were consistent with the size of SCA variances (**Supplementary Table 2**). Incorporating dominance effects improved the prediction accuracy considerably for convergent parent populations, where dominance generated major contributions of SCA effects to the genetic variance among inter-population hybrids (Technow et al., 2012). In Pop2, the AD model didn’t not improve predictive performance in comparison to the A model in most circumstances because the GCA variances (additive variances) was large. High level of additive variances also explained the reason that the A model achieved high accuracies in Pop2. In line with our findings, Ferrão et al. (2020) demonstrated that the inclusion of dominance effects increased the predictive ability of grain yield because dominance explained a large portion of the phenotypic variance for grain yield; when the additive variance was large, the A model yielded better results for grain moisture. The superiority of GBLUP-AD and Gaussian kernel regression depended on the level of dominance variance in sorghum (Ishimori et al., 2020). In addition, the loss in accuracy that was induced by the inclusion of dominance or epistatic effects was most likely caused by more pronounced interactions of environments with dominance and epistatic effects than with additive effects (Liu et al., 2017b).

In most instances, RKHS did not improve the prediction accuracies when the dominance effect was included especially in Pop2. RKHS *per se* can capture non-additive effects in hybrid populations (Gianola et al., 2006), even if only additive genomic matrix was fitted into the method. For trait *per se*, RKHS gave consistently high predictive performance than some parameter models (Ma and Cao, 2021). However, our findings showed that this method was not good for GCA prediction. Alves et al. (2019) found that although RKHS was in all cases the one that had the highest proportion of variance explained, the predictive performance of this model was not the highest one.

The prediction accuracy (0.62-0.64) of GCA effects for GYP in Pop2 based on 11,734 SNPs was higher than that (0.49-0.55) achieved using 39,659 SNPs from the DArT-seq platform (Zhang et al., 2022). For hybrid prediction, high prediction accuracy (0.74-0.75) was achieved for GYP using 11,734 SNPs in Pop2. In some previous studies, lower accuracies (0.03-0.67) were achieved for GYP or grain yield per hectare in hybrid populations with higher marker densities (21,475-52,811) which were obtained from the genotyping-by-sequencing, 50K Illumina chip, maize 500k Affymetrix chip, and Affymetrix genotyping array of 616 K SNPs platforms (**Supplementary Table 3**, Dias et al., 2018; Alves et al., 2019; Schrag et al., 2019; de Oliveira et al., 2020; Dias et al., 2020; Ferrão et al., 2020; Costa-Neto et al., 2021). Using fewer markers, a moderate accuracy was achieved with the AD model for hybrid GYP in Pop1, which was comparable to that achieved in some previous studies (**Supplementary Table 3**). Heffner et al. (2010) concluded that GS can significantly accelerate genetic gains through shortening the breeding cycle if moderate selection accuracies are obtained. Several studies showed that GBTS can significantly reduce the cost of genotyping by at least half compared with the array-based and genotyping-by-sequencing platforms (Guo et al., 2019; Bernardo et al., 2020; Guo et al., 2021). All these indicated that the GBTS-based 5.5K loci panel is sufficient for predicting GCA effects and hybrid performance and will be a reliable, efficient, and low-cost genotyping platform for GS-assisted breeding in selecting high GCA lines and high-yielding hybrids in maize.

## Data availability statement

The data presented in the study are deposited in the Sequence Read Archive repository, accession number PRJNA728476, PRJNA649667, PRJNA681326, PRJNA843481, and PRJNA843784, and in the China National GeneBank DataBase Sequence Archive repository, accession number CNP0003317.

## Author contributions

JM designed the study, collected the phenotypic data, analyzed the phenotypic and genomic data including population structure and genomic prediction analyses, wrote and revised the manuscript. JM, YC, YW, and YD provided the DNA resequencing and RNA sequencing data for GBTS panel development. All authors contributed to the article and approved the submitted version.

## Funding

This work was supported by the Science and Technology Project of Henan Province (222102110043) and by the Science-Technology Foundation for Outstanding Young Scientists of Henan Academy of Agricultural Sciences (2020YQ04).

## Acknowledgments

We would like to thank Dr. Jiankang Wang, Institute of Crop Sciences, Chinese Academy of Agricultural Sciences (CAAS) for providing valuable suggestions for this manuscript.

## Conflict of interest

The authors declare that the research was conducted in the absence of any commercial or financial relationships that could be construed as a potential conflict of interest.

## Publisher’s note

All claims expressed in this article are solely those of the authors and do not necessarily represent those of their affiliated organizations, or those of the publisher, the editors and the reviewers. Any product that may be evaluated in this article, or claim that may be made by its manufacturer, is not guaranteed or endorsed by the publisher.
