# Supplementary material for: Development of the maize 5.5K loci panel for genomic prediction through genotyping by target sequencing
Source: Front Plant Sci. 2022 Nov 11;13:972791. doi: 10.3389/fpls.2022.972791 (PMC9691890; doi:10.3389/fpls.2022.972791)
Supplement: Supplementary file 1 [file DataSheet_1.pdf]

Supplementary Material

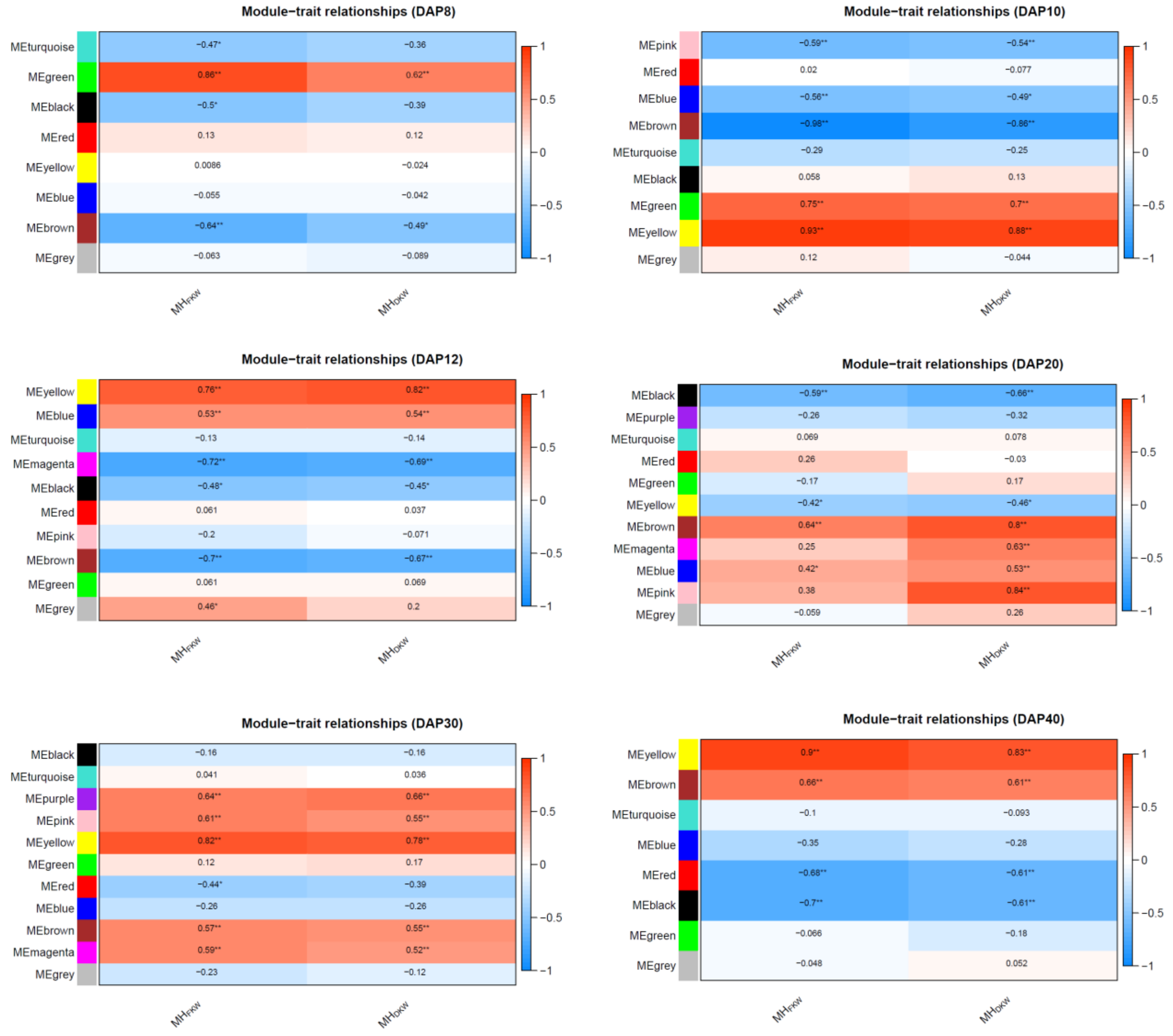

Supplementary Figure 1. The module-trait relationships revealed by weighted gene co-expression network analysis. In each developmental stage, differentially expressed genes between F_1 hybrids and their parents are used to constructed co-expression network. The eigen values of each module represented by colors are correlated with mid-parent heterosis of fresh kernel weight (FKW) and dry kernel weight (DKW). Significant modules with Pearson correlation coefficients > 0.5 are selected for the GBTS-based 5.5K loci panel design. The detailed information of materials and experiment design can be found in a previous study (Wang et al., 2022). DAP represents days after pollination. * and ** represent the Pearson correlation coefficients at significance level of 0.05 and 0.01, respectively.

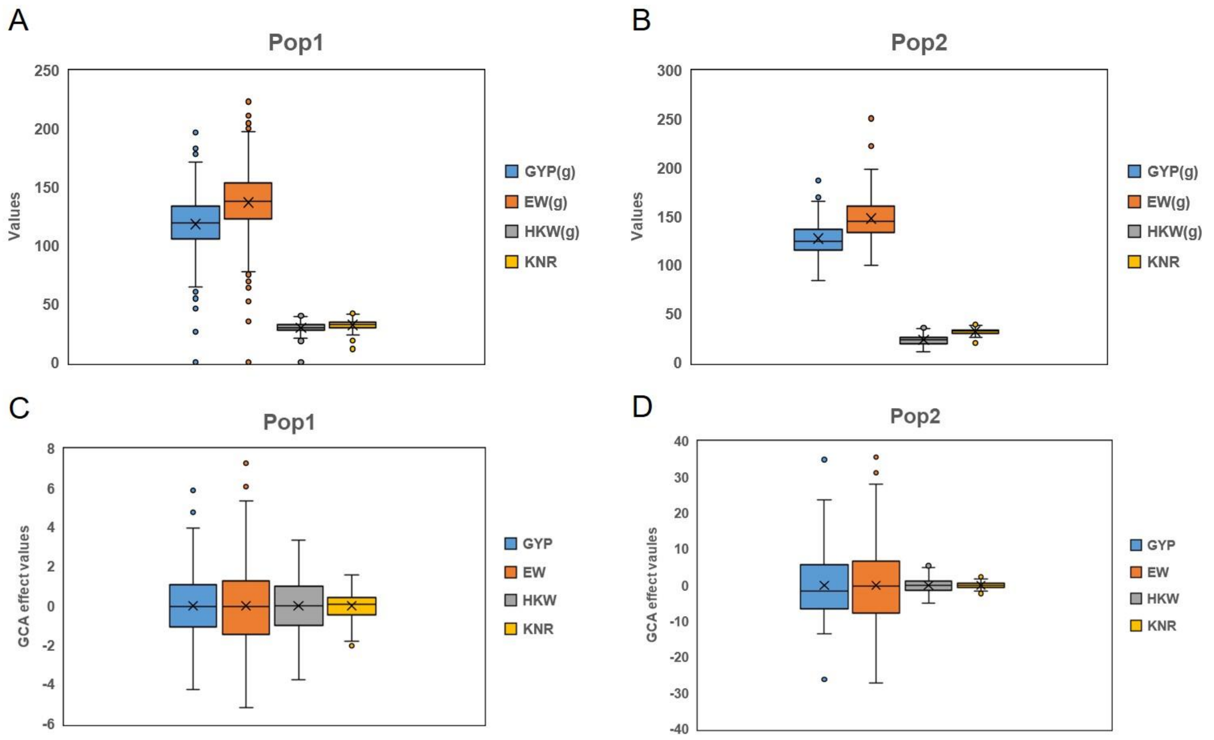

Supplementary Figure 2. Descriptive statistics of hybrid performance and effect values of general combining ability for four traits in two populations. **(A)** and **(B)** represent distributions of hybrid performance in Pop1 and Pop2, respectively. **(C)** and **(D)** represent distributions of effect values of general combining ability in Pop1 and Pop2, respectively. GYP, EW, HKW, and KNR are abbreviations of grain yield per plant, ear weight, thousand-kernel weight, and kernel row number, respectively. × represents means.

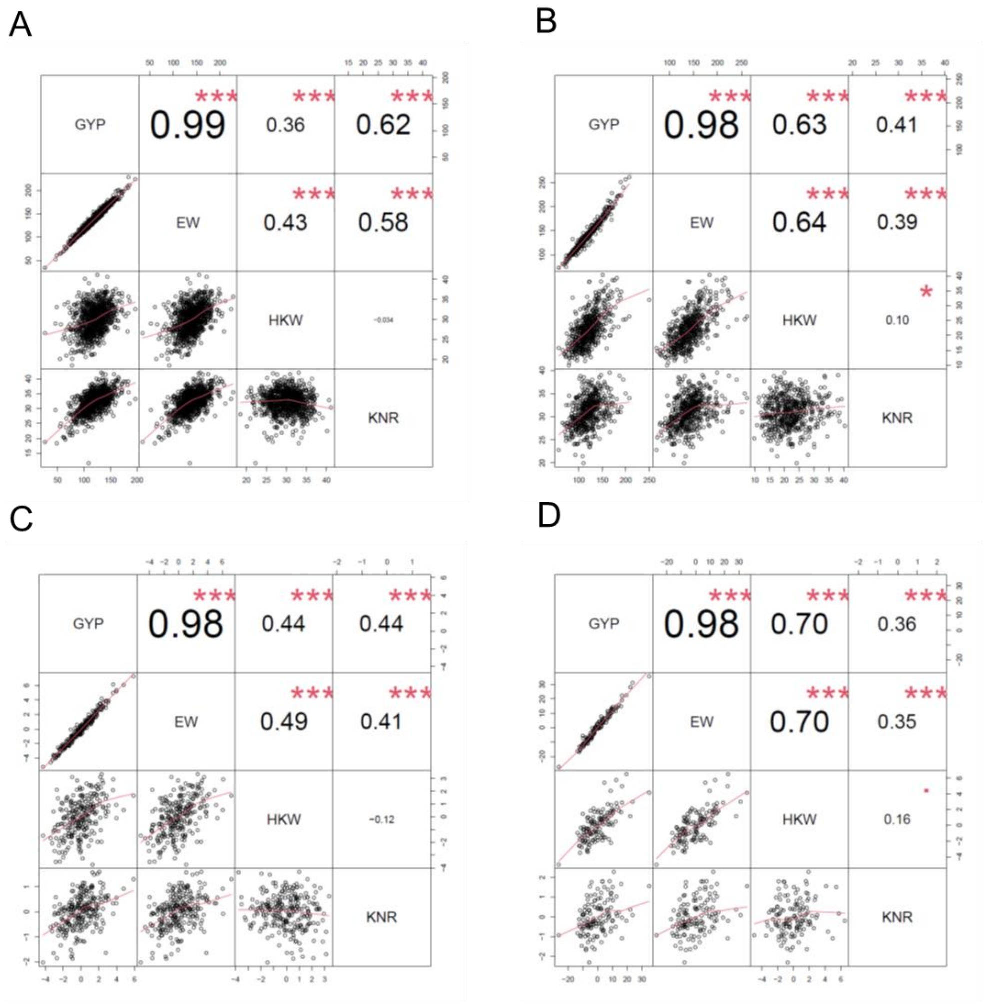

Supplementary Figure 3. Pairwise correlations among four traits and effect values of general combining ability in two populations. **(A)** Pairwise correlations for hybrid traits in Pop1. **(B)** Pairwise correlations for hybrid traits in Pop2. **(C)** Pairwise correlations for effects of general combining ability in Pop1. **(D)** Pairwise correlations for effects of general combining ability in Pop2. GYP, EW, HKW, and KNR are abbreviations of grain yield per plant, ear weight, thousand-kernel weight, and kernel row number, respectively. * and *** represent the Pearson correlation coefficients at significance level of 0.05 and 0.001, respectively.

A

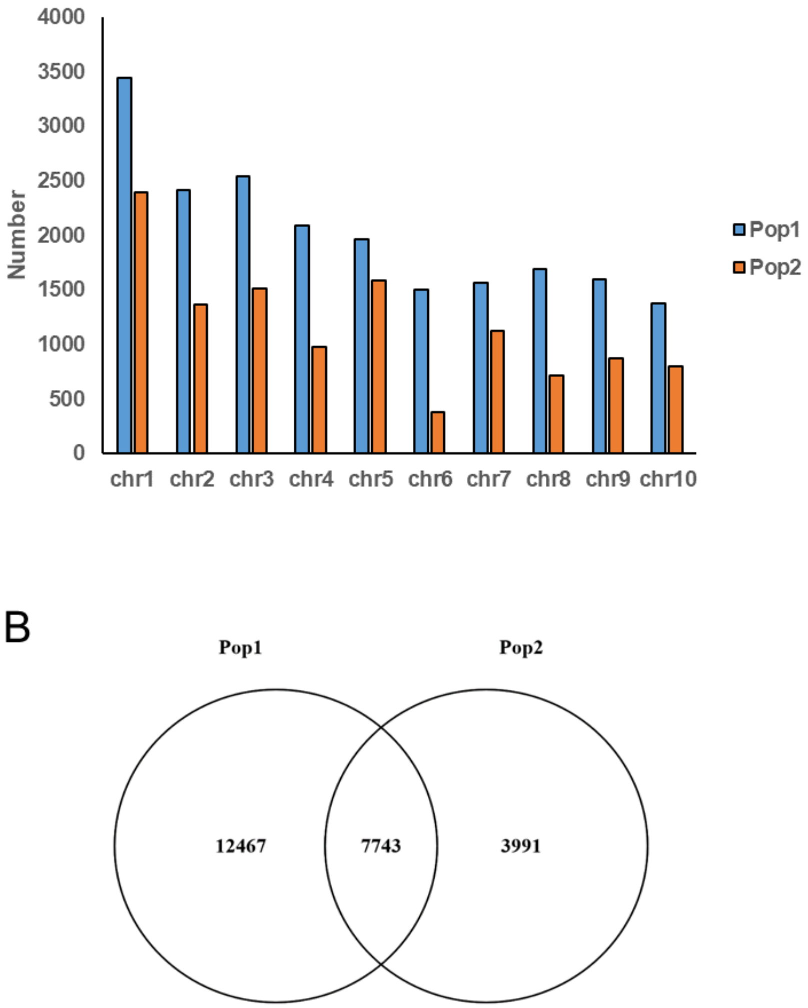

Supplementary Figure 4. (A) The number of filtered SNP markers on ten chromosomes in two populations. **(B)** The venn plot of filtered SNP markers between two populations.

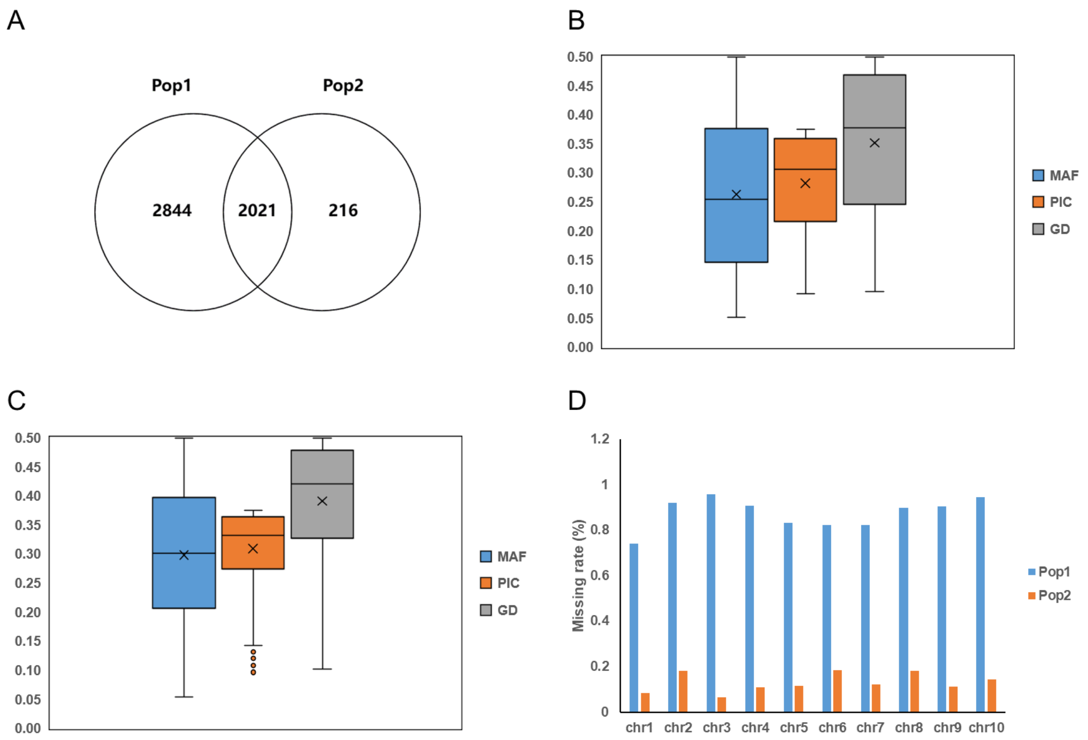

Supplementary Figure 5. The information of target SNPs in two populations. **(A)** The venn plot of target SNPs in both populations. **(B)** and **(C)** represent the distributions of minor allele frequency (MAF), polymorphic information content (PIC), and gene diversity (GD) in Pop1 and Pop2, respectively. **(D)** The missing rate of target SNPs on ten chromosomes in both populations.

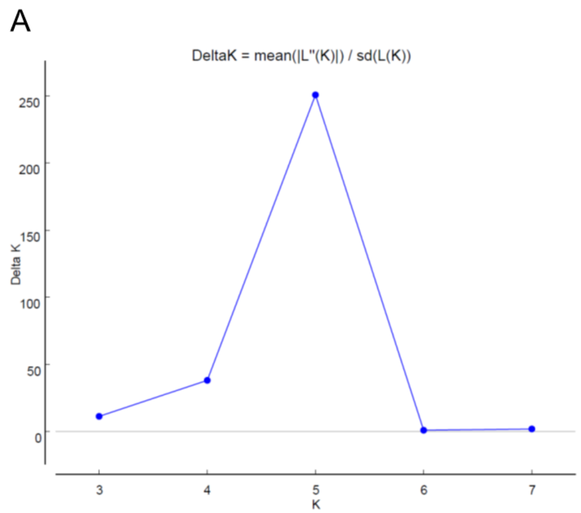

Supplementary Figure 6. Delta K plots derived from the genotypic data of two populations. **(A)** 254 maize inbred lines. **(B)** 131 maize inbred lines.

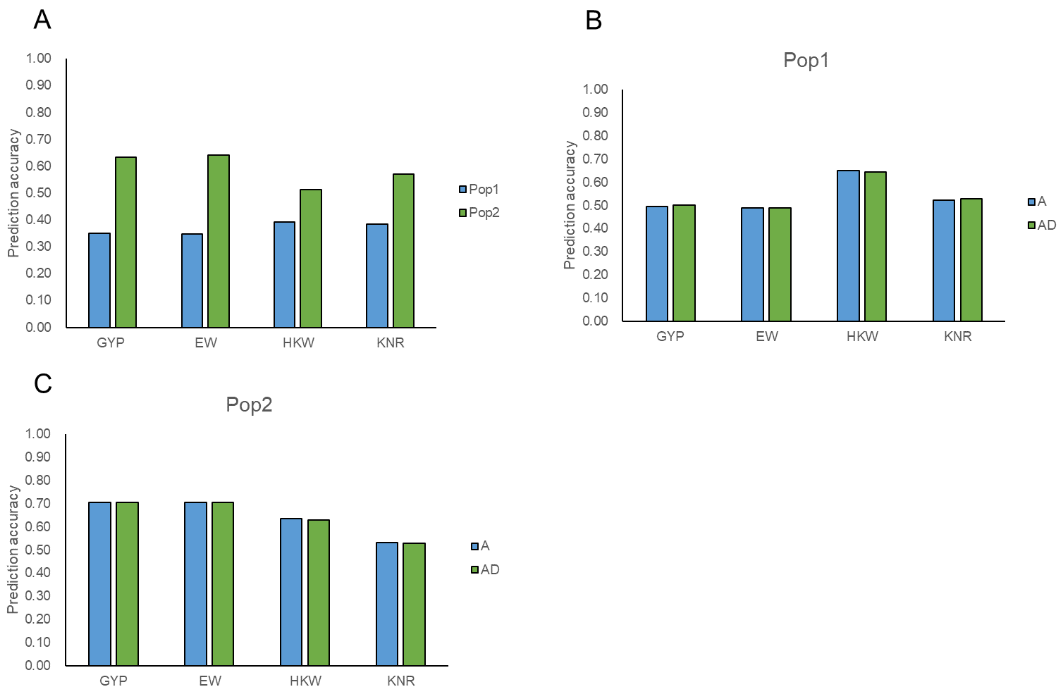

Supplementary Figure 7. Accuracy of 7,743 common SNPs in two populations. **(A)** represents general combining ability prediction in both populations using RKHS method. **(B)** and **(C)** represent hybrid performance prediction using RKHS with additive (A) and additive-dominance model (AD) in Pop1 and Pop2, respectively.

Supplementary Table 1 Information of 131 known genes

Gene ID	Gene name
Zm00001d046449	A9
Zm00001d037057	aaap47
Zm00001d010697	aas12
Zm00001d020208	ABCA7
Zm00001d019951	ABCG38
Zm00001d047359	abi38
Zm00001d017618	ABI3-VP1-transcription factor 16
Zm00001d013722	ABI3-VP1-transcription factor 5
Zm00001d027939	ABI3-VP1-transcription factor 7
Zm00001d040787	abscisic acid stress ripening9
Zm00001d037537	Alfin-like-transcription factor 1
Zm00001d018869	ao5
Zm00001d019734	AP2-EREBP-transcription factor 177
Zm00001d028524	AP2-EREBP-transcription factor 4
Zm00001d007840	AP2-EREBP-transcription factor 41
Zm00001d038394	apk1
Zm00001d042267	ARF-transcription factor 10
Zm00001d053819	ARF-transcription factor 16
Zm00001d014377	ARF-transcription factor 18
Zm00001d014723	ARR-B-transcription factor 5
Zm00001d048705	benzoxazinone synthesis5
Zm00001d021988	bHLH-transcription factor 15
Zm00001d033870	bHLH-transcription factor 27
Zm00001d052192	bHLH-transcription factor 36
Zm00001d007311	bHLH-transcription factor 41
Zm00001d015618	brown midrib1
Zm00001d020122	burp7
Zm00001d010667	bZIP-transcription factor 108
Zm00001d015743	bZIP-transcription factor 61
Zm00001d046751	bZIP-transcription factor 70
Zm00001d020025	bZIP-transcription factor 9
Zm00001d040455	C2C2-Dof-transcription factor 8
Zm00001d025953	C2C2-GATA-transcription factor 6
Zm00001d018829	C2C2-YABBY-transcription factor 3
Zm00001d026543	c3h52
Zm00001d014705	C3H-transcription factor 329
Zm00001d010956	C3H-transcription factor 37
Zm00001d027392	cals9
Zm00001d018255	CCAAT-HAP2-transcription factor 213
Zm00001d036436	cep4
Zm00001d018669	cka4
Zm00001d046356	ckx9
Zm00001d044973	colored aleurone1
Zm00001d045735	conz1

Zm00001d002662	cyclin8
Zm00001d038087	dwarf & irregular leaf1
Zm00001d036880	ebf1
Zm00001d011876	elm1
Zm00001d016260	ERF
Zm00001d025050	etr2
Zm00001d009292	fl3
Zm00001d019536	G2-like-transcription factor 15
Zm00001d033310	G2-like-transcription factor 23
Zm00001d026542	G2-like-transcription factor 52
Zm00001d015407	G2-like-transcription factor 53
Zm00001d022266	GeBP-transcription factor 12
Zm00001d048574	GNAT-transcription factor 39
Zm00001d052112	GRF-transcription factor 10
Zm00001d018260	growth-regulating factor9
Zm00001d016164	GW2
Zm00001d012439	H4C7
Zm00001d037529	hagtf28
Zm00001d037305	hak24
Zm00001d050100	his2b2
Zm00001d032316	Homeobox-transcription factor 112
Zm00001d013547	Homeobox-transcription factor 37
Zm00001d053151	Homeobox-transcription factor 6
Zm00001d034433	HSF-transcription factor 11
Zm00001d046299	HSF-transcription factor 28
Zm00001d052194	hsp22
Zm00001d028408	hsp26
Zm00001d024903	hsp90
Zm00001d045660	kcs22
Zm00001d035194	lug8
Zm00001d019213	MADS-transcription factor 13
Zm00001d013792	MADS-transcription factor 64
Zm00001d031625	MADS-transcription factor 77
Zm00001d020384	milkweed pod1
Zm00001d037926	miniature seed6
Zm00001d019670	mmp187
Zm00001d008251	MYB
Zm00001d039185	MYB_related
Zm00001d024625	mybr84
Zm00001d008808	MYB-related-transcription factor 24
Zm00001d024810	MYB-related-transcription factor 33
Zm00001d010967	MYB-related-transcription factor 85
Zm00001d022628	MYB-transcription factor 11
Zm00001d019712	MYB-transcription factor 153
Zm00001d017243	MYB-transcription factor 86
Zm00001d035084	NAC-transcription factor 123
Zm00001d008403	NAC-transcription factor 130

Zm00001d051140	NAC-transcription factor 17
Zm00001d006053	NAC-transcription factor 24
Zm00001d049443	ocl5a
Zm00001d008939	pebp14
Zm00001d011158	phd10
Zm00001d034074	pho1
Zm00001d018024	PIN-formed protein2
Zm00001d036319	prc1
Zm00001d006451	SBP-transcription factor 13
Zm00001d009646	scl1
Zm00001d035960	smk7
Zm00001d010662	snrkb1
Zm00001d014119	sus6
Zm00001d033673	tb1
Zm00001d053179	tubtf3
Zm00001d004139	upl2
Zm00001d011413	WRKY-transcription factor 28
Zm00001d012516	WUSCHEL-related homeobox transcription factor 13b
Zm00001d028217	Zea mays MADS14
Zm00001d005757	ZF-HD-transcription factor 20
Zm00001d005726	ZIM-transcription factor 15
Zm00001d019228	zip8

Supplementary Table 2 Analysis of variance and heritability for four traits in two populations

Populations	Sources	GYP	EW	HKW	KNR
Pop1	GCA_L	6.42**	6.44**	11.34**	4.41**
	GCA_T	30.51**	29.63**	362.27**	78.55**
	SCA	4.64**	4.49**	3.38**	2.91**
	Environment	167.40**	107.91**	20.24**	190.08**
	Replicate	7.61**	8.39**	0.01	0.42
	GCA_L:Environment	2.92**	2.86**	2.22**	1.71**
	GCA_T:Environment	18.82**	23.96**	87.97**	0.54
	SCA:Environment	2.10**	2.01**	1.99**	1.70**
	σ^2_{GCA}	21.75	29.93	4.94	2.45
	σ^2_{SCA}	201.47	250.18	2.14	4.13
	H^2_{GCA}	0.16	0.17	0.72	0.50
	H^2_{hybrid}	0.64	0.64	0.71	0.60
Pop2	GCA_L	10.88**	10.14**	71.75**	6.22**
	GCA_T	168.17**	139.94**	778.14**	43.15**
	SCA	2.33**	2.31**	19.39**	2.59**
	Environment	2007.44**	2638.64**	7991.23**	127.98**
	Replicate	1.42	2.08	0.14	0.79
	GCA_L:Environment	3.64**	3.66**	16.65**	2.84**
	GCA_T:Environment	10.58**	12.42**	87.52**	9.77**
	SCA:Environment	2.17**	2.42**	21.86**	2.06**
	σ^2_{GCA}	304.72	407.20	12.52	2.74
	σ^2_{SCA}	7.36	0.00	0.00	1.27
	H^2_{GCA}	0.84	0.82	0.84	0.67
	H^2_{hybrid}	0.67	0.63	0.65	0.55

GCA_L and GCA_T represent the GCA effects of lines and testers. GYP, EW, HKW, KNR, GCA, and SCA are abbreviations of grain yield per plant, ear weight, thousand-kernel weight, kernel row number, general combining ability, and special combining ability, respectively. H^2_{GCA} and H^2_{hybrid} represent heritability of GCA and hybrid traits, respectively. σ^2_{GCA} and σ^2_{SCA} represent variances of GCA and SCA, respectively. Other values represent mean of square. ** denotes 0.01 significant level.

Supplementary Table 3 Comparisons of prediction accuracy for grain yield per plant or hectare of hybrid populations with A and AD model in this study and previous studies.

References	Population size of hybrids	Genotyping platform	Marker density	Prediction accuracy
Dias et al. (2018)	308	genotyping-by-sequencing	47,127	0.03-0.58
Dias et al. (2020)	949	genotyping-by-sequencing	21,475	<=0.54
de Oliveira et al. (2020)	415	genotyping-by-sequencing	-	0.07-0.5
Ferrão et al. (2020)	1,831	maize 500k Affymetrix chip	24,758	0.34-0.67
Schrag et al. (2019)	1,970	Illumina SNP chip MaizeSNP50	37,479	<=0.5
Costa-Neto et al. 2021	247	Affymetrix Axiom Maize Genotyping Array of 616 K SNPs	52,811	0.21-0.57
Technow et al. (2014)	1,254	Illumina MaizeSNP50 BeadChip	35,478	0.75-0.92
Pop1(this study)	942	maize GBTS-based 5.5K loci panel	20,210	0.22-0.52
Pop2 (this study)	540	maize GBTS-based 5.5K loci panel	11,734	0.74-0.75

REFERENCES

Costa-Neto, G., Fritsche-Neto, R., and Crossa, J. (2021). Nonlinear kernels, dominance, and envirotyping data increase the accuracy of genome-based prediction in multi-environment trials. *Heredity* 126, 92–106. doi: 10.1038/s41437-020-00353-1

de Oliveira, A. A., Resende, M. F. R., Ferrão, L. F. V., Amadeu, R. R., Guimares, L., Guimares, C. T., et al. (2020). Genomic prediction applied to multiple traits and environments in second season maize hybrids. *Heredity* 125, 60–72. doi: 10.1038/s41437-020-0321-0

Dias, K. O. D. G., Gezan, S. A., Guimarães, C. T., Nazarian, A., Silva, L. C., Parentoni, S. N., et al. (2018). Improving accuracies of genomic predictions for drought tolerance in maize by joint modeling of additive and dominance effects in multi-environment trials. *Heredity* 121, 24–37. doi: 10.1038/s41437-018-0053-6

Dias, K. O. G., Piepho, H. P., Guimares, L. J. M., Guimares, P. E. O., Parentoni, S. N., Pinto, M. O., et al. (2020). Novel strategies for genomic prediction of untested single-cross maize hybrids using unbalanced historical data. *Theor. Appl. Genet.* 133, 443–455. doi: 10.1007/s00122-019-03475-1

Ferrão, L. F. V., Marinho, C. D., Munoz, P. R., and Resende, M. F. R. (2020). Improvement of predictive ability in maize hybrids by including dominance effects and marker × environment models. *Crop Sci.* 60, 666–677. doi: org/10.1002/csc2.20096

Schrag, T. A., Schipprack, W., Melchinger, A. E. (2019). Across-years prediction of hybrid performance in maize using genomics. *Theor. Appl. Genet.* 132, 933–946. doi: 10.1007/s00122-018-3249-5

Technow, F., Schrag, T. A., Schipprack, W., Bauer, E., Simianer, H., and Melchinger, A. E. (2014). Genome properties and prospects of genomic prediction of hybrid performance in a breeding program of maize. *Genetics* 197, 1343–1355. doi: 10.1534/genetics.114.165860

Wang, Y., Nie, L., Ma, J., Zhou, B., Han, X., Cheng, J., et al. (2022). Transcriptomic variations and network hubs controlling seed size and weight during maize seed development. *Front. Plant Sci*. 13, 828923. doi: 10.3389/fpls.2022.828923
